# Ambulatory Assessment of Psychological and Physiological Stress on Workdays and Free Days Among Teachers. A Preliminary Study

**DOI:** 10.3389/fnins.2020.00112

**Published:** 2020-02-14

**Authors:** Alexander Wettstein, Fabienne Kühne, Wolfgang Tschacher, Roberto La Marca

**Affiliations:** ^1^Department of Research and Development, University of Teacher Education Bern, Bern, Switzerland; ^2^University Hospital of Psychiatry Bern, University of Bern, Bern, Switzerland; ^3^Department of Psychology, Clinical Psychology and Psychotherapy, University of Zurich, Zurich, Switzerland

**Keywords:** teacher stress, cortisol, salivary alpha-amylase, heart rate variability, diurnal rhythm

## Abstract

**Objective:**

Teachers are affected by high levels of job stress, leading to one of the highest rates of burnout. The purpose of our pilot study was to investigate the diurnal course of teachers’ psychological and physiological stress responses [cortisol levels, alpha-amylase, heart rate (HR), and heart rate variability (HRV)]. Another aim of the project was to test the applicability of ambulatory assessment methods in daily teaching situations.

**Methods:**

In a non-clinical sample of eight primary school teachers (mean age = 43, *SD* = 15.22, 6 females) in Switzerland, continuous biopsychological data on two workdays and a free day were assessed. The teachers’ HRs and HRV were measured continuously using an ambulatory ECG. Additionally, eight saliva samples were collected from the teachers repeatedly throughout the day to determine the diurnal course of salivary cortisol and alpha-amylase (sAA). Perceived stress and anger ratings were assessed simultaneously.

**Results:**

As hypothesized, the teachers’ morning cortisol levels, perceived stress, and anger levels were significantly higher, and their overall HRV was significantly lower on workdays than on a free day. Conversely, sAA levels and HRs showed no significant differences between working and free days. Salivary markers exhibited the expected diurnal course, with decreasing cortisol and increasing sAA levels over the course of the day, while self-rated stress reached the maximum at midday during working days.

**Conclusion:**

The results of the present explorative study show that physiological and psychological parameters differ within working and free days for teachers. A comparison between working and free days resulted in differences in morning cortisol levels, HRV as well as stress and anger levels. The ambulatory assessment method was found to be applicable in daily teaching situations.

## Introduction

Work-related stress and its impact on health is a major contemporary challenge (Eurofound and EU-OSHA, 2014) in modern societies. In Europe, 25% of workers report work-related stress for most of their time spent working (Eurofound and EU-OSHA, 2014). In Switzerland, the number of chronically stressed employees increased from 27% in 2000 to 34% in 2010 (Ramaciotti and Perriard, 2000; Grebner et al., 2011). Regarding the economic burden in Switzerland, the State Secretariat for Economic Affairs (SECO) estimated that in 2000, stress resulted in costs of over 4.2 billion Swiss francs in the overall working population (Ramaciotti and Perriard, 2000), with increasing numbers in the following years (Grebner et al., 2011).

Work-related stress appears to be especially high in the education sector (Aloe et al., 2014; Eurofound and EU-OSHA, 2014). Occupational stress among teachers has risen dramatically over the past decade, and compared to other professions, burnout is presumed to be highest among teachers (Aloe et al., 2014). Between 20 and 30% of teachers report that being a teacher is either very stressful or extremely stressful (Kyriacou, 2015). In the US, 46% of teachers reported high daily stress during the school year (Gallup, 2014). In Switzerland, similar results were found, with one third of teachers (5^th^ through 9^th^ grade) reporting feeling very stressed (Kunz Heim et al., 2014). Symptoms of exhaustion, fatigue, headache, and tension, as well as mental and psychosomatic diseases, are overrepresented in teachers compared to other professionals (Scheuch et al., 2015). Teachers’ emotional exhaustion further affects their teaching quality and reduces student achievement and student motivation (Klusmann et al., 2016). As another consequence of work-related stress, many teachers leave their profession or retire early (Ingersoll, 2001; Friedman, 2006). Turnover is especially high among new teachers, with 40 to 50% leaving the profession within 5 years (Alliance for Excellent Education, 2017). It is therefore essential to understand and prevent teacher stress.

When we perceive a situation as threatening and when we do not perceive that we have the resources to cope with obstacles, we experience stress. Following Lazarus and Folkman (1984), stress is not an external event itself, but rather a result of the interpretation of a potential threat. For a psychosocial situation to be stressful, it must be appraised as such (Lazarus, 1966). Lazarus and Folkman (1984) postulated that stress appraisal consists of two aspects. Primary appraisal refers to the evaluation of potential threat in terms of the situational demands and the goals and values that we bring into the situation, while secondary appraisal refers to the evaluation of the resources we have at our disposal for dealing with the demands of the situation. Lazarus and Folkman (1984) focus on the stress response as an outcome of a cognitive process. The psychological stress response is reflected by negative affect or feeling nervous and anxious, and the most challenging stressful experiences are accompanied by physiological stress responses (Campbell and Ehlert, 2012).

The autonomic nervous system (ANS) and the hypothalamic-pituitary-adrenal (HPA) axis are two major systems that respond to stressors (Pinel, 2007). The ANS is involved in the regulation of the inner milieu of the organism. During stress situations, the activity of the sympathetic branch increases, while the activity of the parasympathetic branch decreases, leading to alterations in the activity of innervated organs (e.g., increased cardiac activity). Stress-related responses are instrumental in preparing the body for fight-or-flight responses, in contrast to the rest-and-digest response during relaxing moments. At the same time, the HPA axis regulates the release of cortisol, which influences many bodily functions such as metabolic, psychological, and immunological functions.

An overwhelming number of physiological processes are not experienced consciously (Pennebaker, 1982). It is, therefore, important to assess stress not only through self-ratings but also by physiological measurements.

Overall, psychological and physiological responses prepare the organism to deal with perceived challenges and are, therefore, adaptive. When individuals are chronically exposed to stressors, however, it can lead to the development of illnesses, such as depression or cardiovascular diseases. A meta-analysis by Stalder et al. (2017) showed that chronic stress was associated with increased hair cortisol levels. Thus, teachers may experience unhealthy physiological alterations without recognizing this potential risk.

Psychological stress responses are usually measured using self-report measures (Wilhelm and Grossman, 2010; Campbell and Ehlert, 2012), whereas physiological stress reactivity is frequently assessed via the reactivity of the HPA axis and the ANS. The HPA axis, a homeostatic system that follows a circadian rhythm, is activated in response to cognitive-emotional (e.g., fear, excitement, anxiety) or somatic (e.g., infections) stressors (Campbell and Ehlert, 2012). Cortisol, which is the end product of the HPA axis, is one of the most prominent markers assessed in stress research. Cortisol was found to be especially sensitive to anticipated or actual stressful challenges (Rothland and Klusmann, 2012). In a typical diurnal HPA-axis regulation pattern, cortisol levels rise within 20–25 min of awakening and then gradually decline throughout the day. Salivary alpha-amylase (sAA) is one of the most common salivary proteins and reflects stress-related changes in the ANS (Nater et al., 2007). SAA reacts strongly to psychosocial stressors and is not directly related to other physiological stress markers such as cortisol, noradrenalin, or heart rate (HR) (Nater et al., 2003, 2006).

Because cardiac activity is largely under the control of the ANS, HR and heart rate variability (HRV) are often used as physiological stress markers of the ANS. HR reflects the number of contractions (beats) of the heart per minute (Pinel, 2007). HRV represents the variance of the time intervals between successive heartbeats. Stress situations usually cause increases in HR and decreases in vagally-related HRV indices. Therefore, alterations of HR and HRV can be used as indicators of stress (Task Force, 1996).

Research on teacher stress relies mostly on data collected via questionnaires (Krause et al., 2013). While questionnaires seem to be a suitable method for assessing affective stress experiences, they cannot capture actual physiological stress responses in real life (Hellhammer and Schubert, 2012). Little is known about physiological correlates of affective stress in teachers (Rothland and Klusmann, 2012).

One of the few studies on HPA-axis activity in teachers found significantly higher cortisol levels on two working days compared to a leisure day (Bellingrath et al., 2008). The authors stated that the effect was mainly driven by differences in the morning samples. The finding of a stronger cortisol awakening response (CAR) could reflect the anticipation effects of the upcoming day’s demands (Kunz-Ebrecht et al., 2004). In another study, the impact of self-reported job strain on cortisol levels was examined. Teachers experiencing high job strain had significantly higher cortisol levels in the first sample of the day, before the start of schoolwork, compared to teachers with low job strain. No differences were found in other measurements during the day (Steptoe et al., 2000). In line with other researchers, the authors argued that this probably reflected an anticipatory psychobiological response before work. Other evidence for anticipatory cortisol stress responses in teachers was found in a study by Bickhoff (2002). He showed that teachers’ cortisol levels increased during cognitive engagement with upcoming classroom situations, while teaching itself did not have a strong impact on cortisol levels. In addition, the teachers’ cortisol levels at the end of class in the afternoon still differed significantly from their cortisol levels on free days. Bickhoff (2002) ascribed this finding to prolonged cognitive engagement with problems from the school day after class.

Few researchers have examined the cardiovascular activity of teachers. Ritvanen et al. (2006) compared the HRs of female teachers at three selected measurement points during work periods of perceived high and low stress in a slightly artificial situation. HR was always measured in the same sitting position after a resting period. While no difference between high and low stress work periods was found in older teachers, HR before work, during work, and in the evening was significantly higher in younger teachers. According to the authors, this difference reflects the shift to a relative predominance of sympathetic nervous system activity during high stress, which is seen less in older people. A first attempt to assess teachers’ cardiovascular activity in real-life situations goes back to Scheuch and Knothe (1997). They found significantly higher HRs in teachers in teaching compared to organizational duties. Particularly high HRs were found before classes. However, HRV was not assessed. Schwerdtfeger et al. (2008) recorded cardiovascular activity for 22 h on a typical school day for teachers. Results indicated significantly higher HRs during school compared to during leisure time. In contrast, no significant differences were found in HRV between school and leisure time.

The present study goes beyond previous studies. First, it combines cardiovascular measurements in real-life situations with the recording of cortisol and alpha-amylase. Second, it assesses both resources and strains from a long term perspective as well as feelings of stress in specific working conditions. Third, it aims to assess stress among teachers not only at selected measurement points but continuously over the whole day. The selected multidimensional and continuous ambulatory assessment strategy permits an ecologically valid assessment of teacher stress in real-life working situations and enhances our understanding of the complex interplay between psychological, physiological, and social factors.

The objective of our explorative study was to test the applicability of an ambulatory assessment approach for recording the diurnal course of teacher stress on two working days and one free day in real life. Whereas in medical science physiological methods are routinely employed, they are rarely applied in pedagogical-psychological research. In the current study, we pursued two goals. First, we wished to better understand teachers’ psychological stress and related physiological alterations in the work environment. Based on the few studies higher CARs, higher HRs and lower HRVs on workdays than on the free day are expected. Given the paucity of previous research, we had no clear assumptions regarding the direction of differences in alpha-amylase secretion between workdays and the free day. For the psychological stress response, we expected higher stress and anger levels on the workdays compared to the free day. Having a healthy sample of teachers, we hypothesized an increase in cortisol levels after awakening and a decrease thereafter, while an opposite circadian course for alpha-amylase was expected (see for example Nater et al., 2007; Steptoe and Serwinski, 2016). The diurnal course of the psychological stress reaction should be examined in an exploratory manner. A positive association between CAR self-rated work overload is predicted (see review by Chida and Steptoe, 2009). Second, we intended to examine the applicability of ambulatory assessment methods in daily teaching situations.

## Materials and Methods

### Participants

To recruit participants, we contacted the principals of primary schools in the canton of Bern (Switzerland), who informed their teams. The teachers contacted the research team directly. The inclusion criterion was that participants teach at least 16 lessons per week. Exclusion criteria, besides smoking and pregnancy, were drug or substance abuse, repeated use of cardiovascular drug and other medication in the past 2 months, consumption of psychoactive substances in the last 4 weeks, more than two standard alcoholic drinks per day, long-haul flights the last 2 weeks, acute infections, and cardiovascular or other chronic diseases. All teachers were screened in a short interview in order to ensure that the inclusion criteria were met. Of 11 teachers who consented to participate in the pilot project, three had to be excluded due to smoking (*n* = 2) and pregnancy (*n* = 1), resulting in a final sample of eight teachers (six females; mean age = 43.00 years, *SD* = 15.22, range = 25–62). On average they had 17.5 years of teaching experience (*SD* = 15.41, range = 1–39). Participants were not compensated for their participation in the study.

### Procedures and Design

**FIGURE 1:**
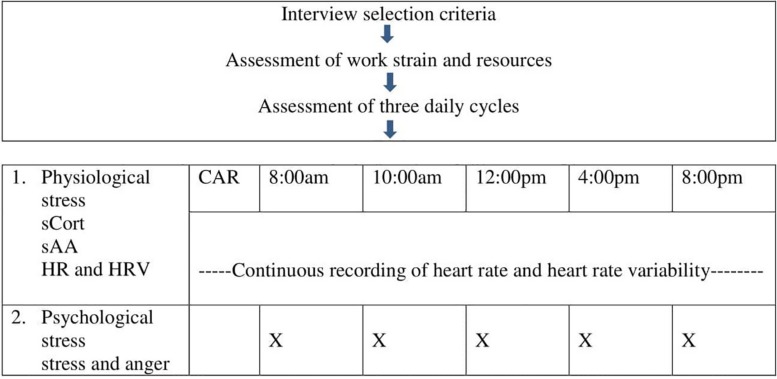
Study design. sCort, salivary cortisol; CAR, cortisol awakening response after awakening, 30 and 45 min later; sAA, salivary alpha-amylase; HR, heart rate; HRV, heart rate variability.

At a first appointment, the goal of the study was explained to the participants, and written informed consent was obtained from all of the teachers. Subsequently, the teachers’ work strain was assessed (Figure 1). Participants received a summary with important information, including a “do not” list (e.g., abstaining from alcoholic beverages, avoiding excessive physical exercise for 18 h prior to the study day) before taking part in the study. The procedure and the use of collection devices were explained and practiced. Data collections were performed on the subsequent three days, from awakening time until 8:00 pm. Two measurement days were workdays with normal work schedules and one was a free day. In order to assess the diurnal course of cortisol and alpha-amylase (sAA), the teachers collected eight saliva samples on each of the measurement days between awakening and 8:00 pm. At each measurement point, affective stress experiences were rated by the teachers using paper-pencil questionnaires.

### Measures

#### Biochemical Measures

On each measurement day, eight saliva samples were collected using cotton rolls (Salivettes, Sarstedt, Sevelen, Switzerland). The subjects were asked to refrain from heavy physical exercise and drinking alcohol 48 h beforehand, as well as to avoid physical exertion and caffeine (e.g., coffee, coke, energy drinks, black tea) on the day of the study examination. Participants were also asked not to eat or drink, to chew gum and to use a toothbrush 2 h prior to study participation. Subjects were instructed to chew the cotton rolls for 1 min immediately after waking up, 30 and 45 min later, and at 8:00 am, 10:00 am, 12:00 pm, 4:00 pm, and 8:00 pm. The first sample was taken while still lying in bed. After the collection, the samples were stored in the participants’ own refrigerators prior to storage in the study refrigerator at −20°C. After completion of the data collection, biochemical analyses were conducted at the biochemical laboratory of the department of Clinical Psychology and Psychotherapy at the University of Zurich. Salivary cortisol levels were analyzed using an immunoassay with time-resolved fluorescence detection (Dressendörfer et al., 1992), while the activity of sAA was determined using a kinetic colorimetric test (see Boesch et al., 2014).

#### Electrophysiological Measures

Heart rate variability measurement is sensitive to methodological aspects (Laborde et al., 2017). For the assessment of HRV, we followed the guidelines of the Task Force on HRV (Task Force, 1996). Electrocardiogram (ECG) data were captured with the EcgMove3 sensor from Movisens Version 1.9.31.0 (movisens GmbH, Karlsruhe, Germany), which is easy to apply. The sensor records a single channel ECG at a sampling rate of 1024 Hz. After awakening, the teachers attached the sensor with adhesive electrodes. Thereafter, ECG data were collected until 8:00 pm. The sensor also measures the physical activity based on the registration of acceleration in three dimensions and atmospheric air pressure. Using this data we compared segments within and between subjects with a similar physical activity level.

### Activity Diary and Self-Ratings

For each measurement day (two workdays, one free day), the subjects kept a diary to assess their activities. In addition, during each saliva collection, the subjects filled out two 11-point visual analog scales (VAS) to rate how stressed and angry they had felt during the previous hour. Furthermore, arousal experienced during the past hour was reported on a nine-point Self-Assessment Manikin (SAM), a non-verbal graphic rating system (SAM; Lang, 1980). On the Manikin, the arousal dimension is represented by a relaxed sleepy figure at one end of the scale and an excited, wide-eyed figure at the other end. Subjects could additionally indicate whether there was something that had stressed them during the past hour.

#### Work Strain and Resources

Demographic variables (gender, age, years of teaching experience, number of lessons taught per week, number of students in the filmed class) were obtained once from each teacher. In addition, they answered various trait questionnaires, of which only the Trier Inventory of Chronic Stress (TICS; Schulz et al., 2004) was relevant for the hypotheses in question. The TICS consists of 57 items covering nine factors of chronic psychosocial stress (work discontent, work overload, pressure to succeed, excessive demands, social surcharge, social tensions, social isolation, lack of social recognition, and chronic worrying). The items assessing the frequency of stressful events in the past 3 months were rated on a five-point Likert scale (“never” to “very often”). The TICS has been broadly used in stress research and shows satisfactory internal consistency (Cronbach’s alpha was between 0.84 and 0.91, depending on the scale) and validity (Schulz et al., 2004).

### Data Analyses

For ECG data processing and analysis, Kubios HRV Premium version 3.0.2 was used (Niskanen et al., 2004). The raw data were edited manually in Kubios. After artifact correction, the data were exported into SPSS. HRV analysis can be conducted in the time and in frequency domains. In order to assess the long-term components of HRV, we calculated the overall values for SDNN, SDANN, HRV triangular, and RMSSD (Task Force, 1996).

The data analysis took place in a within-subjects design (Laborde et al., 2017). Due to the small sample size (*N* = 8), two-tailed non-parametric analyses of variance (ANOVAs) for repeated measures were computed using Friedman’s ANOVA (Friedman, 1937) to test possible time effects. For *post hoc* tests, Dunn-Bonferroni tests with Bonferroni correction for multiple testing were performed. One-tailed non-parametric correlations between physiological and psychological measures were computed using Kendall’s Tau (Field, 2013). In order to compare physiological and psychological measures at different time points, two-tailed Wilcoxon signed-rank tests were calculated. For cortisol and alpha-amylase, the area under the total response curve with respect to the ground (AUCg) and with respect to increase (AUCi) were calculated for each of the three measurement days using the trapezoid formula (Pruessner et al., 2003). Means for the two workdays were computed. Data were analyzed using SPSS Statistics 23 (IBM; Armonk, NY, United States), and *p*-values of < 0.05 were considered to be statistically significant.

## Results

### Physiological Stress Responses

On workdays, each participant collected the 16 saliva samples (eight per day) as planned. On the free day, a total of nine saliva samples at 8:00 am and 10:00 am were missing due to late awakening times, resulting in a total of 183 samples. In the ECG data, there was one missing free day for one participant due to an error in reading the ECG.

### The Diurnal Course of Salivary Cortisol and Alpha-Amylase Levels

**FIGURE 2:**
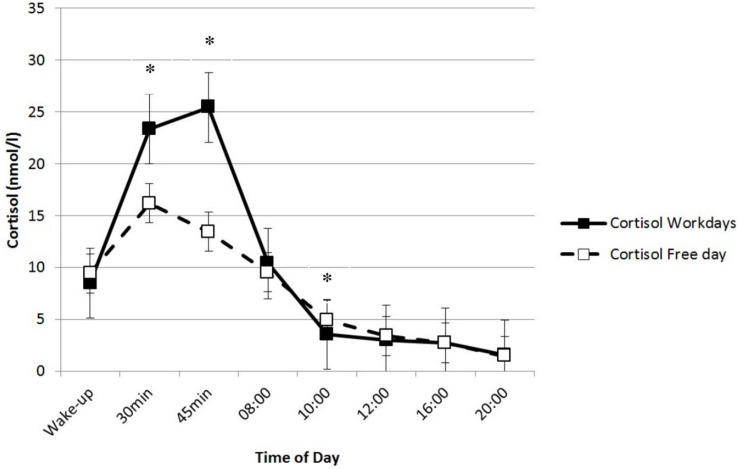
The diurnal course of cortisol responses. The asterisks mark significant differences in cortisol release on working days and on days off.

Cortisol exhibited the expected diurnal course on workdays and the free day (Figure 2), with a peak after awakening (CAR) and a decrease thereafter [work days: chi-square(7) = 52.83, *p* < 0.01; free day: chi-square(6) = 30.93, *p* < 0.01]. *Post hoc* tests revealed significantly higher cortisol levels in the first morning sample compared to the last sample of the day at 8:00 pm (*z* = 3.67, *p*_*adjusted*_ < 0.01) on workdays. On the free day, the cortisol levels at 8:00 pm did not differ significantly from the first morning sample right after awakening (*z* = 2.94, *p*_*adjustedt*_ = 0.069).

**FIGURE 3:**
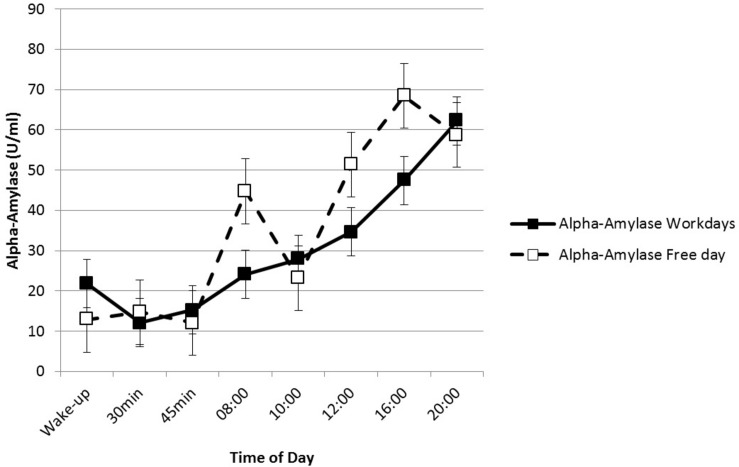
The diurnal course of alpha-amylase responses.

Salivary alpha-amylase changes over time were significant on both work and free days [workdays: chi-square(7) = 35.65, *p* < 0.01; free day: chi-square(6) = 20.71, *p* < 0.01]. SAA levels decreased significantly within the first hour of awakening and increased toward the afternoon and evening (Figure 3). On workdays, levels of sAA were significantly lower 30 min after awakening compared to the levels at 10:00 am (*z* = −3.42, *p*_*adjusted*_ = 0.018), 12:00 pm (*z* = −3.16, *p*_*adjusted*_ = 0.044), 4:00 pm (*z* = −3.39, *p*_*adjusted*_ < 0.01), and 8:00 pm (*z* = −4.64, *p*_*adjusted*_ < 0.01). SAA levels on the free day showed a similar course with significantly higher sAA levels in the last sample of the day (8:00 pm) compared with the first morning sample (*z* = −3.34, *p*_*adjusted*_ = 0.018), 30 min after awakening (*z* = −3.21, *p*_*adjusted*_ = 0.028), and 45 min after awakening (*z* = −3.07, *p*_*adjusted*_ = 0.044). Thus, the expected diurnal patterns were confirmed.

### Physiological Differences on Workdays and the Free Day

Wilcoxon signed-rank tests were performed to examine the differences in salivary cortisol and alpha-amylase secretion (sAA), HR, and HRV between the workdays and free day. Further effect sizes (r) were calculated.

Results indicated no significant difference in cortisol levels right after awakening on workdays (median = 8.47) compared to the free day (median = 9.41; *z* = 0.14, *p* = 0.89, *r* = 0.04). Cortisol secretion 30 min after awakening was significantly higher on workdays (median = 23.38) than on the free day (median = 16.19; *z* = −2.52, *p* = 0.012, *r* = −0.67). The same was true for 45 min after awakening (median work days = 25.42, median free day = 13.45; *z* = −2.52, *p* = 0.012, *r* = −0.67). Differences in AUC values further supported these findings: the CAR was significantly higher on workdays (median AUCg = 821.84; median AUCi = 441.25) than on the free day (median AUCg = 607.78; median AUCi = 147.38; AUCg: *z* = −2.52, *p* = 0.012, *r* = −0.67; AUCi: *z* = −2.52, *p* = 0.012, *r* = −0.67). At 10:00 am, however, cortisol levels were lower on workdays (median = 3.53) compared to the free day (median = 4.93; *z* = −2.20, *p* = 0.028, *r* = −0.59). No statistically significant differences were found for other measurement time-points. For sAA, no significant differences on workdays or the free day were found (all tests n.s.). However, if one considers corrections for multiple tests with a Bonferroni test, none of the reported results reaches statistical significance.

Heart rate did not differ significantly between workdays (median = 79.15) and the free day (median = 77.14; *z* = −1.18 *p* = 0.24, *r* = −0.32). Concerning the long-term components of HRV, the SDNN was significantly lower on workdays (median = 96.23) compared to the free day (median = 134.98; *z* = 2.20, *p* = 0.028, *r* = 0.59). SDANN showed the same pattern being significantly lower on workdays (median = 73.39) than on the free day (median = 109.64; *z* = 2.20, *p* = 0.028, *r* = 0.59). A comparison of the HRV triangular index on workdays and the free day failed to reach statistical significance (median workdays = 29.18, median free day = 40.95; *z* = 1.86, *p* = 0.063, *r* = 0.50). The same was true for RMSSD; RMSSD on workdays (median = 23.70) was lower than on the free day (median = 28.66) but was not significant (*z* = 1.69 *p* = 0.091, *r* = 0.45).

### Psychological Stress

#### The Diurnal Course of Perceived Stress and Anger

Friedman’s ANOVA was calculated to detect differences in perceived stress and anger. On workdays, *perceived stress* showed an increase in the morning, peaking at 12:00 pm, followed by a decrease until the evening [chi-square(4) = 16.58, *p* < 0.01]. On the free day, levels of perceived stress did not differ significantly across different measurement time-points [chi-square(3) = 2.00, *p* = 0.572]. Levels of perceived stress did not differ significantly across different measurement time-points.

In terms of *anger* (Visual Analog Scale) the results revealed significant differences on workdays [chi-square(4) = 11.17, *p* = 0.025], peaking at 12:00 pm, with *post hoc* comparisons showing no significant pairwise differences (all *n* = n.s.). Anger measured with the SAM showed a significant result on workdays [chi-square(4) = 18.97, *p* < 0.01], with maximum anger at 12:00 pm. Dunn-Bonferroni-tests showed significantly higher anger levels at 12:00 pm than at 8:00 pm (*z* = 4.11, *p*_*adjusted*_ < 0.01). On the free day, no significant differences in anger levels between the different time-points were detected [Visual Analog Scale: chi-square(3) = 3.00, *p* = 0.39; SAM: chi-square(3) = 3.67, *p* = 0.300].

#### Perceived Stress and Anger on Workdays and Free Days

Wilcoxon signed-rank tests were conducted to determine whether there was a difference in perceived stress and anger on workdays versus the free day. Further effect sizes (r) were calculated.

A comparison of *perceived stress* at 10:00 am on workdays and the free day showed significantly higher levels of perceived stress on workdays (median = 2.50) than on the free day (median = 0.00; *z* = −2.03, *p* = 0.042, *r* = −0.54). Similar results were observed in the measurement at 12:00 pm (median workdays = 3.25; median free day = 0.00; *z* = −2.54, *p* = 0.011, *r* = −0.68) and 4:00 pm (median workdays = 1.50; median free day = 0.00; *z* = −2.21, *p* = 0.027, *r* = −0.59), a time point where teachers were not teaching anymore. No statistically significant difference in perceived stress was found for the last measurement time-point at 8:00 pm (median workdays = 0.00; median free day = 0.00; *z* = 0.37, *p* = 0.715, *r* = 0.10).

Similar to perceived stress, *anger* measured with the Self-Assessment-Manikin (SAM) differed significantly at 10:00 am, with higher levels of anger on workdays (median = 2.75) than on the free day (median = 1.00; *z* = −2.04, *p* = 0.041, *r* = −0.55). Also, at 12:00 pm (median workdays = 5.00; median free day = 1.00) and 4:00 pm (median workdays = 2.75; median free day = 1.00), anger was higher on workdays than on the free day (12:00 pm: *z* = −2.55, *p* = 0.011, *r* = −0.68; 4:00 pm: *z* = −2.38, *p* = 0.018, *r* = −0.64). Measurement of anger using the VAS yielded comparable results with significantly higher levels of anger at 12:00 pm on workdays (median = 2.50) than on the free day (median = 0.00; *z* = −2.21, *p* = 0.027, *r* = −0.59), while other measurement time-points did not differ significantly between workdays and the free day. If one considers corrections for multiple tests with a Bonferroni test, none of the reported results reaches statistical significance.

#### Associations Between Self-Rated Work Overload and Physiological Stress Markers

The relations between physiological stress markers and self-reported chronic work stress (measured using the TICS; Schulz et al., 2004) were tested using non-parametric correlations (Table 1). General dissatisfaction at work was significantly correlated with an increased cortisol CAR on workdays. Social tensions were significantly associated with increased AUCi on workdays, but not on the free day. Lack of social recognition was significantly correlated with AUCg on the free day and the workdays.

**TABLE 1:** The association between general work stress and cortisol awakening response.

	**Cortisol awakening response (CAR)**			
	**Working days**		**Free day**	
**General work stress**	**AUCg**	**AUCi**	**AUCg**	**AUCi**
Work discontent	0.62*	0.69**	0.33	–0.11
Work overload	0.40	0.11	0.40	0.40
Pressure to succeed	0.18	0.04	0.18	0.04
Excessive demands	0.42	–0.19	0.34	–0.11
Social surcharge	0.00	0.00	–0.15	0.22
Social tensions	0.54*	0.77**	0.46	0.22
Social isolation	0.27	0.34	0.11	0.41
Lack of social recognition	0.56*	0.56*	0.65*	0.40
Chronic worrying	0.15	0.00	0.07	0.07

AUCg = area under the total response curve with respect to the ground, AUCi = area under the curve with respect to increase. Values correspond to Kendall’s tau coefficient. * *p* < 0.05, ** *p* < 0.01 (one-tailed).

## Discussion

The primary aim of the current study was to investigate the diurnal course of psychological and physiological stress responses in a sample of eight healthy primary school teachers using ambulatory assessment methods and to determine the differences between workdays and a free day. Cortisol and alpha amylase (sAA) showed the expected diurnal course with decreasing cortisol and increasing sAA levels. Results also revealed that the teachers’ morning cortisol and perceived stress and anger levels were significantly higher on workdays than on the free day, whereas overall HRV was significantly lower on workdays compared to the free day. SAA, RMSSD, and HR, in contrast, showed no significant differences between workdays and the free day.

*Salivary cortisol* showed a typical circadian pattern with higher levels in the morning and lower evening levels. This result is in line with previous findings (Steptoe and Serwinski, 2016). For *alpha-amylase* (sAA), we found a marked diurnal profile with a pronounced decrease in the first 30 min after awakening and steadily rising levels toward the afternoon and evening, which is typical for the circadian course of sAA (Nater et al., 2007).

Teachers showed a clearly higher CAR on workdays compared to a free day. This is consistent with the findings of other studies. For example, Kunz-Ebrecht et al. (2004) reported that in British civil servants, CAR was significantly higher on workdays than on weekend days. Similarly, Schlotz et al. (2004) found significantly higher CAR on weekdays compared to weekends. The CAR is a part of the circadian cortisol rhythm that occurs in most healthy people (Pruessner et al., 1997; Wuest et al., 2000). Since its initial systematic description (Pruessner et al., 1997), researchers have speculated about the functional role of the CAR (Powell and Schlotz, 2012). One established explanation is formulated in the anticipation hypothesis (Schulz et al., 1998; Schlotz et al., 2004). Referring to the role of cortisol in the provision of energy, this hypothesis proposes that the CAR helps prepare the organism for coping with the demands of the upcoming day. Because teaching is considered a stressful occupation (Aloe et al., 2014), higher CAR on workdays compared to the free day in our study could be interpreted within the framework of the anticipation hypothesis and reflects cognitive engagement with upcoming workday activities.

The differences in CAR may also be triggered by differences in physical activity on workdays compared to the free day, assuming that teachers are more active just immediately after waking up on workdays. Research has suggested that moderate exercise has no significant influence on the cortisol reaction (see, for example, Kirschbaum and Hellhammer, 1994); thus, this explanation seems unlikely but cannot be fully ruled out. There is also evidence for an influence of awakening time and sleep duration on the CAR (Edwards et al., 2001; Kudielka and Kirschbaum, 2003), with earlier awakening being associated with a greater increase, whereas individuals waking up later show a smaller increase. Yet results are inconsistent in this respect. Other studies have found no influence of time of awakening on the CAR (see, for example, Pruessner et al., 1997; Hucklebridge et al., 1999; Wuest et al., 2000). Therefore, the influence of awakening time on the CAR on workdays in our sample cannot be fully clarified.

In the present study, we found lower cortisol levels at 10:00 am on workdays compared to the free day. This unexpected result may be attributed to different awakening times. As the teachers woke up earlier on workdays, at 10:00 am their cortisol levels had already decreased. We found no difference in cortisol secretion right after awakening. Thus, the differences in the CAR between workdays and the free day cannot be traced back to a higher cortisol level at the first measurement point. Overall, empirical findings show a clear weekend–weekday difference in the cortisol response to awakening (CAR), with higher cortisol levels on weekdays compared to weekend days. A pronounced CAR on workdays might reflect the anticipation effects of upcoming everyday demands.

For sAA, we found no significant differences in the mean levels on workdays and the free day. Previous findings (Nater et al., 2007) indicated that the daily course of sAA levels might instead reflect chronic stress and stress reactivity than momentary perceived stress. Likewise, Out et al. (2013) found no significant influence of momentary and moderate levels of stress on circadian levels of sAA. Significant alterations in the diurnal rhythm of sAA may be more likely to occur in response to chronic stress. A different picture emerges when focusing on the momentary levels of sAA. In fact, Nater et al. (2007) assessed sAA at hourly intervals and demonstrated that these momentary measures were highly sensitive to acute physical and psychological stress.

For HR, we found no significant differences between mean levels on workdays and the free day. Cardiac activity also changes due to processes that are not stress-related (Zanstra and Johnston, 2011). The missing difference in HR between workdays and the free day could also be attributed to more overall physical activity on the free day, and overall, HR might be aligned to the level of the (stressful) workdays. Analysis of the teachers’ self-reports in their diaries shows that many of them did housework, like cleaning or moderate exercise (cycling, walking), on their free day (data not presented), so this explanation might apply.

However, the teachers’ HRV was significantly lower on workdays compared to the free day. This result is in line with previous findings, which demonstrated that work stress is associated with reduced HRV (Hanson et al., 2001; Riese et al., 2004; Collins et al., 2005; Uusitalo et al., 2011). HRV reflects the capacity to adapt to changing internal and external demands (Porges, 1995). Lower HRV may therefore be the result of a relatively lower vagal tone on workdays. Reduced HRV might hamper social interactions in the classroom. Teachers must adapt their behavior in the classroom rapidly to the dynamic nature of emerging social situations. Reduced HRV might reduce teachers’ capacity for adaptive emotional responding and coping strategies.

In sum, we found significant differences in HRV but not HR between workdays and the free day. HRV is predominantly under the influence of the parasympathetic nervous system. It can be hypothesized that the teachers in our sample only experienced moderate levels of stress, such that effects of the parasympathetic nervous system on cardiovascular activity were mainly observed. In contrast, HR is also under the control of the sympathetic system and remains largely unaffected.

### Psychological Stress

On workdays, the diurnal course of perceived stress showed an increase during the morning, peaking at 12:00 pm, followed by a decrease until the evening. Teaching is a highly demanding task. Teachers have to create opportunities for active learning and simultaneously must structure complex social situations in the classroom. Teachers act continuously in public, without opportunities to withdraw from the situation and with no real recreational time in the morning. Thus, the peak in perceived stress and anger at 12:00 pm likely reflects these accumulated demands from the morning. However, all examined teachers showed good recovery until 8:00 pm. In contrast to workdays, on the free day, levels of perceived stress did not differ significantly across different measurement time-points.

If we compare teachers’ *perceived stress and anger on workdays and free days*, we observe significantly higher levels of perceived stress and anger on the workdays than on the free day, albeit with a complete recovery until 8:00 pm. These results are in line with previous findings (Kunz-Ebrecht et al., 2004) showing that workdays were rated as more stressful, unhappier, and less controllable than weekend days.

Finally, we examined the degree to which physiological stress markers are associated with self-rated *work overload*. We found significant correlations between an increased CAR on workdays and self-reported dissatisfaction at work and social tensions. Previous research showed that work overload is especially critical in association with negative emotions (Schaarschmidt and Fischer, 2008). Lack of social recognition was significantly associated with an increased CAR on the free day, also showing a trend on workdays. The higher association between the CAR on the free day and lack of social recognition could be attributed to the fact that the questions assessing the lack of social recognition (e.g., “I get too little recognition for my achievements.”) were attributed to private areas of life and/or that teachers expect social recognition mostly from their families and friends.

*In sum*, the findings reveal that teachers experience significantly higher stress levels on workdays than on free days. Perceived anger and stress on workdays are also reflected by physiological markers, leading to higher endocrine stress responses and reduced HRV.

### Limitations and Strengths

The present study has several limitations that need to be considered. First, the findings of the present study are based on a small sample of healthy and medication-free teachers and cannot be generalized to the entire population. Second, albeit ambulatory approaches contribute to our understanding of real-life situations (Fahrenberg et al., 1997; Wilhelm and Grossman, 2010; Kockler et al., 2017), they also have definite limitations. Whereas laboratory experiments provide systematic and reliable information on functional relations between particular stimuli and elicit reactions under well-controlled conditions, ambulatory assessment of physiological parameters faces many methodological challenges. In real-life settings, many factors influence physiological state, including posture, physical activity, consumption of food and drinks, circadian rhythms, speech, and social situations (Houtveen and de Geus, 2009). Although ambulatory assessment has better ecological validity than experiments, the lack of experimental control over participants’ posture and physical activity can be a significant source of confounding. In our study, we controlled for physical activity and measurement durations. However, HRV is also affected by respiratory depth, circadian influences, fatigue, mood, and nutrition. Altogether, we think that laboratory and field approaches are complementary and not opposing strategies; they offer data and insight from different angles (Wilhelm and Grossman, 2010).

At the same time, the present study also has significant strengths. Research on teacher stress is usually limited to self-reports, representing retrospective ‘one-shot’ examinations of psychological events. Questionnaires do not do justice to the complexity of the phenomenon of stress. From a biopsychosocial perspective, health and illness – and therefore also stress – are the result of an interplay of biological, psychological, and social factors (Engel, 1977; Ehlert and La Marca, 2017). An essential method of studying psychophysiological stress responses are laboratory experiments conducted under controlled conditions (Wilhelm and Grossman, 2010). Its generalizability, though, is limited. To understand stress in the workplace, it seems to be a more promising strategy, to assess stress in real-life situations with an ambulatory assessment strategy (Vrijkotte et al., 2000; Rau et al., 2001; Fahrenberg et al., 2007; Wilhelm and Grossman, 2010). We assessed teacher stress multidimensionally by measuring several different biological and psychological factors (Engel, 1977; Ehlert and La Marca, 2017) continuously, simultaneously, and in real-life conditions, which opens a new view of everyday school life. The aggregation of different daily situations also results in a robust and ecologically valid measure of teachers’ strain in their daily occupational environments. The inclusion of biochemical and electrophysiological measures, on the one hand, and the assessment of perceived stress and anger and work strains, on the other hand, enhance our understanding of the multifaceted stress processes in teachers.

It is often difficult to clarify whether different data on existing stresses are due to objectively different working conditions or subjectively different perceptions of teachers (Krause et al., 2013). The ambulatory assessment approach makes it possible to investigate which events in very concrete teaching situations trigger a psychological stress experience in teachers and to what extent mental stress is reflected on a biological level. It provides essential information on how stressful experiences are ‘embodied’ in the physiological and endocrine time series.

Using an ambulatory assessment strategy, it is also possible to look at coping patterns of teachers in stress situations in class. It enables us to identify the dynamics of unfavorable coping patterns and escalations in teacher-pupil interactions and to use this knowledge in the prevention of such dynamics. In addition to the use for the classroom development on the class or school level, it also provides teachers on an individual level with valuable information on their strains and resources in their specific work situation and helps them to derive concrete measures for changes in lesson design. A multi-method and ecologically valid recording of burdens and resources in real work situations, therefore, ultimately aims at an improvement of the work situation and, finally also of teaching quality, student motivation, and teacher health.

This present study has corroborated the feasibility of our approach – our research has shown that the tested combination of methods is manageable and allows an ecologically valid assessment of teacher stress in everyday life. The results additionally suggest that the presented assessment method has broad and useful applicability and can be adapted to other areas of stress research, as it enables a continuous and task-related description of work stress in many different environments.

## Data Availability Statement

The raw data supporting the conclusions of this manuscript will be made available by the authors, without undue reservation, to any qualified researcher.

## Ethics Statement

This study was carried out in accordance with the recommendations of the Ordinance on Human Research with the Exception of Clinical Trials. The protocol was reviewed and approved by the Swiss Ethics Committee Bern on research involving humans. All subjects provided written informed consent in accordance with the Declaration of Helsinki.

## Author Contributions

AW and RL designed the research. AW and FK performed the assessments and prepared the first draft. FK, RL, and AW analyzed the data. RL and WT provided insightful comments that critically improved the manuscript quality.

## Conflict of Interest

The authors declare that the research was conducted in the absence of any commercial or financial relationships that could be construed as a potential conflict of interest. The reviewer CB declared a shared affiliation, with no collaboration with the authors to the handling Editor.
